# Phenotype and kinetics of SARS-CoV-2-specific T cells in COVID-19 patients with acute respiratory distress syndrome

**DOI:** 10.1126/sciimmunol.abd2071

**Published:** 2020-06-26

**Authors:** Daniela Weiskopf, Katharina S. Schmitz, Matthijs P. Raadsen, Alba Grifoni, Nisreen M.A. Okba, Henrik Endeman, Johannes P.C. van den Akker, Richard Molenkamp, Marion P.G. Koopmans, Eric C.M. van Gorp, Bart L. Haagmans, Rik L. de Swart, Alessandro Sette, Rory D. de Vries

**Affiliations:** 1 Center for Infectious Disease, La Jolla Institute for Immunology, La Jolla, CA 92037, USA.; 2 Department of Viroscience, Erasmus MC, Rotterdam, the Netherlands.; 3 Department of Intensive Care, Erasmus MC, Rotterdam, the Netherlands.; 4 Department of Pathology, University of California, San Diego, CA 92037, USA.; 5 Department of Medicine, University of California, San Diego, CA 92037, USA.

## Abstract

SARS-CoV-2 has been identified as the causative agent of a global outbreak of respiratory tract disease (COVID-19). In some patients the infection results in moderate to severe acute respiratory distress syndrome (ARDS), requiring invasive mechanical ventilation. High serum levels of IL-6, IL-10 and an immune hyperresponsiveness referred to as a ‘cytokine storm’ have been associated with poor clinical outcome. Despite the large numbers of COVID-19 cases and deaths, information on the phenotype and kinetics of SARS-CoV-2-specific T cells is limited. Here, we studied 10 COVID-19 patients who required admission to an intensive care unit and detected SARS-CoV-2-specific CD4^+^ and CD8^+^ T cells in 10 out of 10 and 8 out of 10 patients, respectively. We also detected low levels of SARS-CoV-2-reactive T cells in 2 out of 10 healthy controls not previously exposed to SARS-CoV-2, which is indicative of cross-reactivity due to past infection with ‘common cold’ coronaviruses. The strongest T-cell responses were directed to the spike (S) surface glycoprotein, and SARS-CoV-2-specific T cells predominantly produced effector and Th1 cytokines, although Th2 and Th17 cytokines were also detected. Furthermore, we studied T-cell kinetics and showed that SARS-CoV-2-specific T cells are present relatively early and increase over time. Collectively, these data shed light on the potential variations in T-cell responses as a function of disease severity, an issue that is key to understanding the potential role of immunopathology in the disease, and also inform vaccine design and evaluation.

## Teaser

Peptide pool stimulation enables longitudinal analysis of SARS-CoV-2-specific CD4^+^ and CD8^+^ T cells in ICU-admitted COVID-19 patients.

## INTRODUCTION

A novel coronavirus named SARS-CoV-2 has been identified as the causative agent of a global outbreak of respiratory tract disease, referred to as COVID-19 (*1*, *2*). COVID-19 is characterized by fever, cough, dyspnea and myalgia (*2*), but in some patients the infection results in moderate to severe acute respiratory distress syndrome (ARDS), requiring invasive mechanical ventilation for a period of several weeks. COVID-19 patients may present with lymphopenia (*2*, *3*), but the disease has also been associated with immune hyperresponsiveness referred to as a ‘cytokine storm’ (*4*). A transient increase in co-expression of CD38 and HLA-DR by T cells, a phenotype of CD8^+^ T-cell activation in response to viral infection, was observed concomitantly (*5*). This increase in both CD4^+^ and CD8^+^ CD38^+^HLA-DR^+^ T cells preceded resolution of clinical symptoms in a non-severe, recovered, COVID-19 patient (*6*).

Despite the large numbers of cases and deaths, there is limited information on the presence and phenotype of SARS-CoV-2-specific T cells, especially in ARDS patients. Spike surface glycoprotein (S)-, membrane (M)- and nucleoprotein (NP)-specific T cells were detected in PBMC from convalescent COVID-19 patients (*7*). More recently, Grifoni *et al*. reported the presence of SARS-CoV-2-specific T cells in convalescent samples from predominantly mild COVID-19 patients. They showed strong reactivity to the viral S and M proteins, and also strong CD4^+^ T-cell responses to N. Additionally, 8 other ORFs were targeted by both CD4^+^ and CD8^+^ T cells (*8*). Virus-specific T cells have also been detected after exposure to the related SARS-CoV and MERS-CoV, although few studies have characterized cellular responses in human patients. For SARS-CoV-specific CD4^+^ T cells it was reported that the S glycoprotein accounted for nearly two-thirds of T-cell reactivity, with N and M also accounting for limited reactivity (*9*). For MERS-CoV-specific CD4^+^ T cells, responses targeting S, N and a pool of M and E peptides have been reported (*10*).

Here, we stimulated peripheral blood mononuclear cells (PBMC) from ten COVID-19 patients with ARDS, collected up to three weeks after admission to the intensive care unit (ICU), with MegaPools (MP) of overlapping or prediction-based peptides covering the SARS-CoV-2 proteome (*11*). We detected SARS-CoV-2-specific CD4^+^ and CD8^+^ T cells in 10/10 and 8/10 COVID-19 patients, respectively. Peptide stimulation of healthy control (HC) age-matched PBMC samples collected before the outbreak in most cases resulted in undetectable responses, although some potential cross-reactivity due to infection with ‘common cold’ coronaviruses was observed. SARS-CoV-2-specific T cells predominantly produced effector and Th1 cytokines, although Th2 and Th17 cytokines were also detected.

## RESULTS

### Patient characteristics

**Fig. 1:**
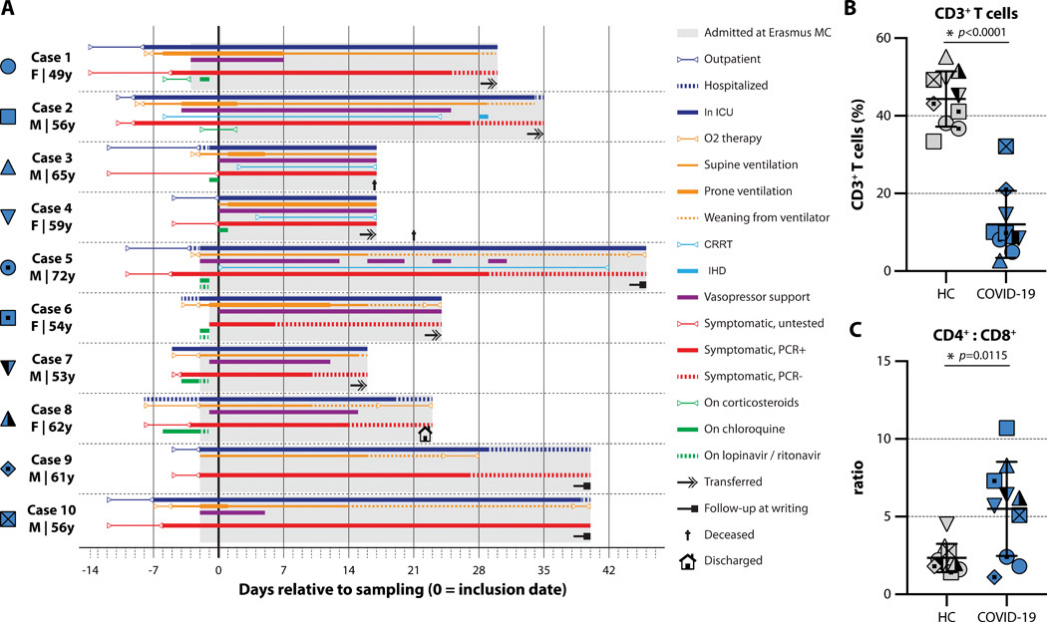
Clinical overview of moderate to severe COVID-19 ARDS patients. (A) Onset of symptoms, hospitalization status, treatment and follow-up of n=10 COVID-19 ARDS patients included in this study. PBMC samples were obtained weekly after admission to the study. Symbols shown next to the cases match throughout all figures. (B) Percentages of CD3^+^ T cells within the total LIVE gate measured by flow cytometry performed on PBMC collected 14 days post inclusion. (C) CD4:CD8 ratios measured by flow cytometry performed on PBMC collected 14 days post inclusion. Panels b and c show individual values for n=10 patients versus n=10 HC, as well as the mean ± SD. Asterisk denotes a significant difference. HC = healthy control.

We included ten COVID-19 patients with moderate to severe ARDS in this study, and compared these to ten age-matched HC. All patients were included in the study shortly after ICU admission; the duration of self-reported illness varied between 5 and 14 days before inclusion (Fig. 1A). Patients were between 49 and 72 years old (average 58.9 ± 7.2 years) and of mixed gender (4 female, 6 male). HC were between 30 and 66 years old (average 43 ± 13.6 years, not statistically different from the patient group) and of mixed gender (4 female, 4 male, no data available for 2 donors). All patients tested SARS-CoV-2 positive by RT-PCR and were ventilated during their stay at the ICU. At the time of writing, 5 patients were transferred out of the ICU (case 1, 2, 4, 6 and 7), 3 patients were still in follow-up (case 5, 9 and 10), 1 patient was discharged (case 8) and 1 patient was deceased (case 3). Case 4 died 4 days after transfer out of the ICU. Patients were treated with lung protective ventilation using the higher PEEP/lower FiO_2_ table of the ARDSnet and restrictive volume resuscitation. They received antibiotics as a part of a treatment regimen aimed at selective decontamination of the digestive tract. Furthermore, all patients received chloroquine, lopinavir-ritonavir and/or corticosteroids for a brief period of time around admission to the ICU (Fig. 1A).

### COVID-19 ARDS patients present with lymphopenia

Phenotyping analysis of PBMC collected 14 days post inclusion via flow cytometry indicated that COVID-19 patients presented with low percentages of CD3^+^ T cells in peripheral blood, corresponding to the previously reported lymphopenia (12.1 ± 8.7% in COVID-19 vs 44.3 ± 7.1% in HC, *p*<0.0001, Fig. 1B) (*2*, *3*). CD4:CD8 ratios were increased in COVID-19 patients when compared to HC (5.5 ± 3.0 in COVID-19 vs 2.3 ± 0.9 in HC, *p*=0.0115, Fig. 1C).

### SARS-CoV-2 peptides and predicted epitopes

PBMC from COVID-19 ARDS patients were stimulated with four different peptide MPs: MP_S, MP_CD4_R and two MP_CD8 pools. MP_S contained 221 overlapping peptides (15-mers overlapping by 10 amino acids) covering the entire S glycoprotein and can stimulate both CD4^+^ and CD8^+^ T cells. MP_CD4_R (R=remainder) contained 246 HLA class II predicted epitopes covering all viral proteins except S, specifically designed to activate CD4^+^ T cells. The two MP_CD8 pools combined contained 628 HLA class I predicted epitopes covering all SARS-CoV-2 proteins, specifically designed to activate CD8^+^ T cells (*11*). Results obtained with MP_CD8_A and MP_CD8_B have been concatenated and shown as a combined stimulation named MP_CD8, but results obtained with separate stimuli are also shown. In addition to stimulation of PBMC from COVID-19 ARDS patients, PBMC from ten HC were tested in parallel. PBMC from healthy controls were obtained before 2020 and could therefore not contain SARS-CoV-2-specific T cells. However, they potentially contain cross-reactive T cells induced by circulating seasonal ‘common cold’ coronaviruses (*12*).

**Fig. 2:**
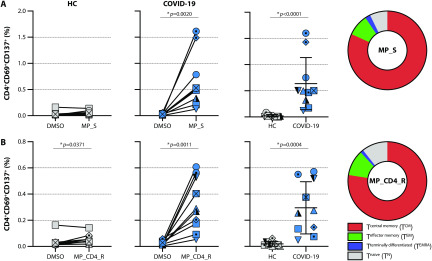
SARS-CoV-2-specific CD4^+^ T-cell responses in COVID-19 ARDS patients. (A, B) Antigen-specific activation of CD4^+^ T cells after stimulation for 20 hours with MP_S (A) and MP_CD4_R (B), measured via cell surface expression of CD69 and CD137 (gating in Fig. S1). Two left panels show activation percentages (within CD3^+^CD4^+^) obtained with the vehicle control (DMSO) and specific stimulation (MP) for HC and COVID-19 patients. The third panel shows the specific activation percentages corrected by subtracting the background present in the DMSO stimulation to allow comparison of both groups. The fourth panel shows the memory phenotype of the CD69^+^CD137^+^ responder cells in a donut diagram. Panels show individual values for n=10 patients versus n=10 HC, as well as the mean ± SD. Asterisk denotes a significant difference. HC = healthy control. Symbol shapes of COVID-19 patients are identical between panels, and refer back to Fig. 1.

**Fig. 3:**
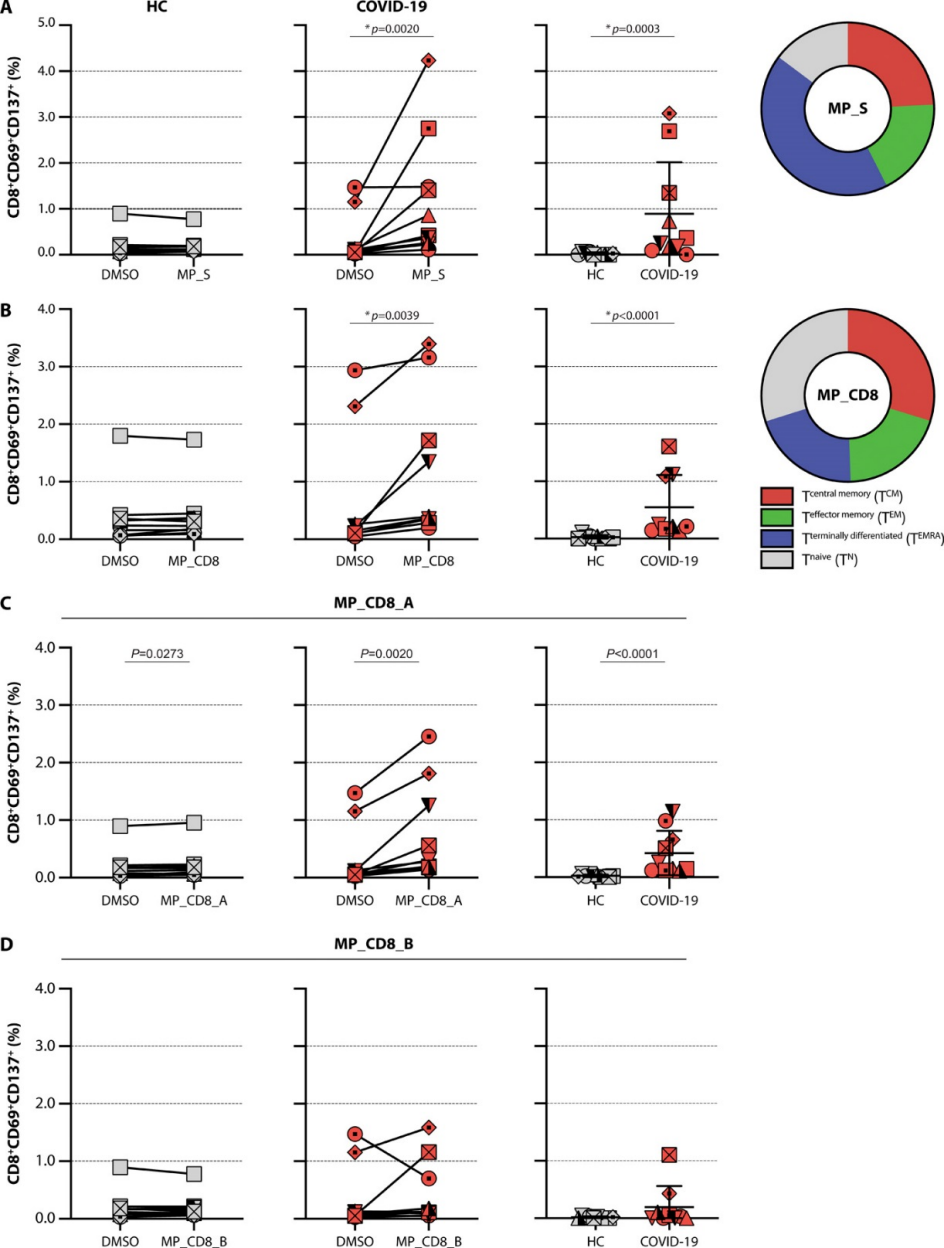
SARS-CoV-2-specific CD8^+^ T-cell responses in COVID-19 ARDS patients. (A, B, C, D) Antigen-specific activation of CD8^+^ T cells after stimulation for 20 hours with MP_S (A), MP_CD8_A (C), and MP_CD8_B, measured via cell surface expression of CD69 and CD137 (gating in Fig. S1). MP_CD8 panels (B) show the concatenated responses from panels C and D. Two left panels show activation percentages (within CD3^+^CD8^+^ gate) obtained with the vehicle control (DMSO) and specific stimulation (MP) for HC and COVID-19 patients. The third panel shows the specific activation percentages corrected by subtracting the background present in the DMSO stimulation to allow comparison of both groups. The fourth panel shows the memory phenotype of the CD69^+^CD137^+^ responder cells in a donut diagram. Panels show individual values for n=10 patients versus n=10 HC, as well as the mean ± SD. Asterisk denotes a significant difference. HC = healthy control. Symbol shapes of COVID-19 patients are identical between panels, and refer back to Fig. 1.

Stimulation of PBMC collected 14 days post inclusion with the different peptide pools led to consistent detection of CD4^+^ and/or CD8^+^ SARS-CoV-2-specific T cells in COVID-19 ARDS patients (Fig. 2, Fig. 3). Specific activation of CD4^+^ and CD8^+^ T cells was measured via cell surface expression of CD69 and CD137; phenotyping of memory subsets was based on surface expression of CD45RA and CCR7 (Fig. S1).

### Characterization of SARS-CoV-2-specific CD4^+^ T cell responses

**Fig. 4:**
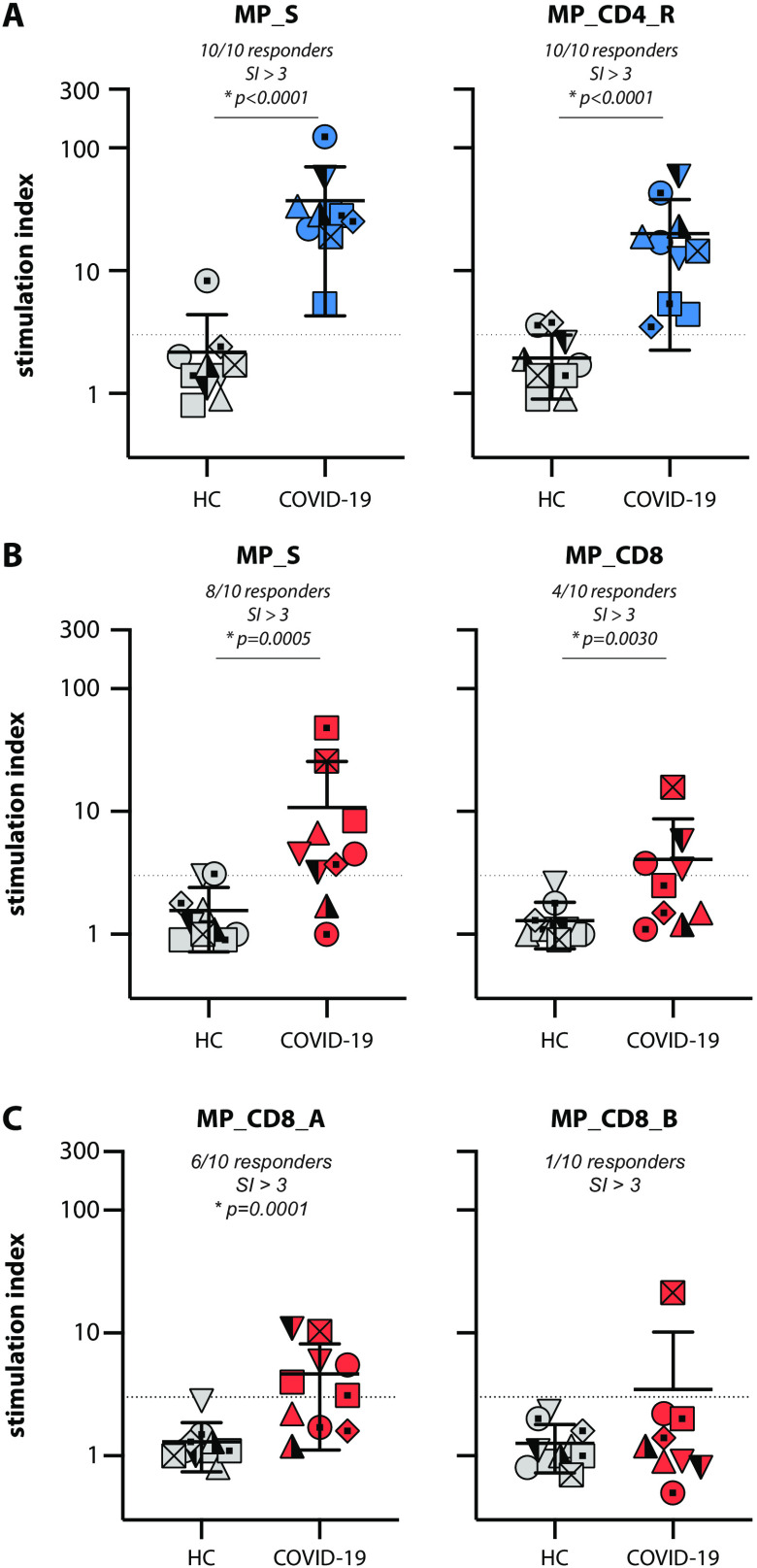
Stimulation index identifies specific responders. Antigen-specific activation of CD4^+^ (A) and CD8^+^ T cells (B,C) in COVID-19 patients after stimulation for 20 hours with peptide MegaPools (MP) shown as stimulation index (SI). Stimulation index is derived by dividing the percentage obtained with specific stimulation (MP) by the percentage obtained with the vehicle control (DMSO). Values for respective stimulations are shown in Fig. 2 (CD4^+^, color coded in blue) and Fig. 3 (CD8^+^, color coded in red). Donors with a SI > 3 (dotted line) are regarded as responders to MP stimulation. Panels show individual values for n=10 patients vs. n=10 HC, as well as the mean ± SD. Asterisk denotes a significant difference. HC = healthy control. Symbol shapes of COVID-19 patients are identical between panels, and refer back to Fig. 1.

Stimulation of PBMC with MP_S and MP_CD4_R led to consistent activation of SARS-CoV-2-specific CD4^+^ T cells (Fig. 2) in PBMC obtained from COVID-19 ARDS patients. Significant responses were detected when activation percentages after stimulation with MP_S and MP_CD4_R were compared with the vehicle control (DMSO). To allow comparison between HC and COVID-19 ARDS patients, we corrected the MP-specific activation percentages by subtracting the value obtained in the DMSO stimulation. Significant T-cell responses were observed in COVID-19 ARDS patients when compared with HC (0.64% in COVID-19 vs 0.02% in HC, *p*<0.0001 for MP_S and 0.29% in COVID-19 vs 0.02% in HC, *p*=0.0004 for MP_CD4_R, Fig. 2A and B, respectively). The stimulation index (SI) was calculated by dividing the MP-specific responses by the DMSO responses, and donors with a SI > 3 were regarded responders (Fig. 4A). According to this definition all COVID-19 ARDS patients responded to the MP_S and MP_CD4_R pools, whereas 1/10 and 2/10 of the HC responded, respectively (Fig. 7). Overall, the MP_S peptide pool induced stronger responses than the MP_CD4_R peptide pool, indicating that the S glycoprotein is a strong inducer of CD4^+^ T-cell responses. Phenotyping of CD4^+^CD69^+^CD137^+^ activated T cells identified the majority of these SARS-CoV-2-specific T cells as central memory T cells, based on CD45RA and CCR7 expression (T_CM_). T_CM_ express homing receptors required for extravasation and migration to secondary lymphoid tissues, but also have high proliferative capacity with low dependence on co-stimulation (*13*, *14*).

**Fig. 5:**
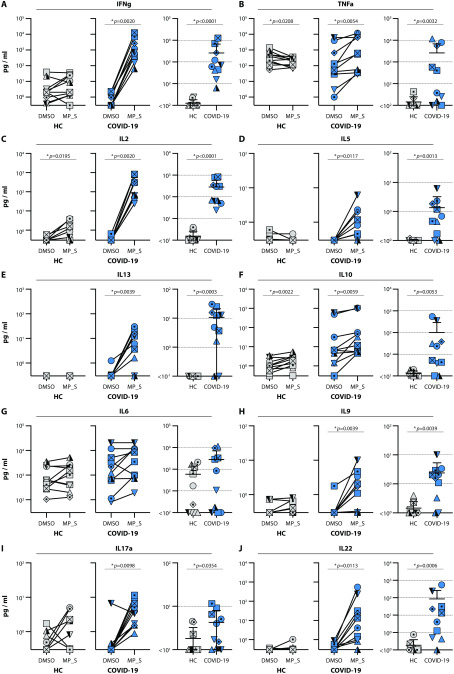
SARS-CoV-2-specific cytokine production in COVID-19 ARDS patients. (A-J) Antigen-specific production of cytokines measured in cell culture supernatants from PBMC obtained 14 days post ICU admission stimulated (20 hours) with MP_S. Two left panels show quantities obtained with the vehicle control (DMSO) and specific stimulation (MP) for HC and COVID-19 patients. The third panel shows the quantity corrected by subtracting the background present in the DMSO stimulation to allow comparison of both groups. Panels show individual values for n=10 patients versus n=10 HC, as well as the geometric mean. Asterisk denotes a significant difference. HC = healthy control. Additional cytokines (IL-4, IL-17F and IL-21) are shown in Fig. S2. Symbol shapes of COVID-19 patients are identical between panels, and refer back to Fig. 1.

**Fig. 6:**
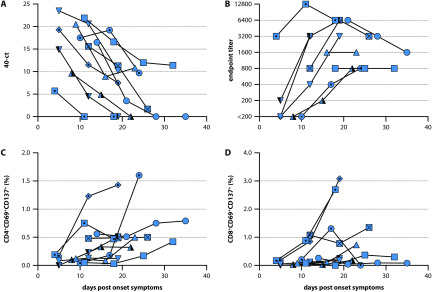
SARS-CoV-2 replication and humoral and cellular immune response kinetics in COVID-19 ARDS patients. (A, B, C, D) Sequential measurements of SARS-CoV-2 genomes detected in upper respiratory tract samples by real-time RT-PCR (40-ct, A), SARS-CoV-2-specific serum RBD IgG antibody levels detected by ELISA (OD450, B) and percentage SARS-CoV-2-specific CD4^+^ and CD8^+^ T cells after MP_S stimulation of PBMC (C, D), plotted against days post onset of symptoms. Genome levels showed a significant decrease over time, antibody levels and specific CD4^+^ T-cell frequencies significantly increased (*p*<0.001, ANOVA repeated measures). A specific increase or decrease of specific CD8^+^ T cells over time was not detected (*p*=0.1001, ANOVA repeated measures). Symbol shapes of COVID-19 patients are identical between panels, and refer back to Fig. 1.

**Fig. 7:**
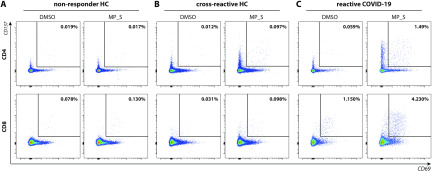
Expression of activation markers in representative samples. (A, B, C) Representative activation plots showing CD69 and CD137 up-regulation from a non-responder HC (A), cross-reactive HC (B) and reactive COVID-19 sample (C). Each panel shows activation after stimulation with DMSO (negative control) or MP_S. Top row shows CD4^+^ T cell responses, bottom row shows CD8^+^ T cell responses. Percentages indicate activated CD69^+^CD137^+^ cells as a fraction of either CD4^+^ or CD8^+^ T cells.

### Characterization of SARS-CoV-2-specific CD8^+^ T cell responses

SARS-CoV-2-specific CD8^+^ T cells were activated by both the MP_S and MP_CD8 peptide pools when compared to vehicle control (Fig. 3A and B). Mainly the peptides pooled in MP_CD8_A were responsible for this activation (Fig. 3C and D). Furthermore, significant responses were detected when activation percentages after stimulation with MP_S and MP_CD8 were compared between HC controls and COVID-19 ARDS patients after DMSO correction (0.90% in COVID-19 vs 0.03% in HC, *p*=0.0003 for MP_S and 0.57% in COVID-19 vs 0.03% in HC, *p*<0.0001 for MP_CD8, Fig. 3A and B). In addition to inducing specific CD4^+^ T cells, the S glycoprotein also induced CD8^+^ T cell responses. Calculation of the SI identified 8/10 and 4/9 (not enough cells were obtained for MP_CD8 stimulation for 1 donor) of the COVID-19 ARDS patients as responders to MP_S and MP_CD8, respectively, whereas 1/10 of the HC responded to the MP_S stimulation (Fig. 4B, C and 7). Phenotyping of CD8^+^CD69^+^CD137^+^ activated T cells showed that these had a mixed phenotype. The majority of virus-specific CD8^+^ T cells was identified as CCR7^-^ effector memory (T_EM_) or terminally differentiated effector (T_EMRA_) (*13*). Both these CD8^+^ effector subsets are potent producers of IFN-γ, contain preformed perforin granules for immediate antigen-specific cytotoxicity and home efficiently to peripheral lymphoid tissues (*14*, *15*).

### Cytokine profiles after antigen-specific stimulation

As production of pro-inflammatory cytokines can be predictive of clinical outcome for other viral diseases (*16*), we measured antigen-specific production of 13 cytokines in cell culture supernatants from PBMC after stimulation. The same samples as shown in Fig. 1-4 were included in this analysis, using samples obtained 14 days after ICU admission. PBMC were stimulated with the respective peptide pools, cytokine production after MP_S stimulation is shown in Fig. 5 and Fig. S2 as representative data. When compared to the vehicle control stimulation, PBMC obtained from COVID-19 ARDS patients specifically produced IFN-γ, TNF-α, IL-2, IL-5, IL-13, IL-10, IL-9, IL-17A, IL-17F and IL-22 after MP_S stimulation (Fig. 5, Fig. S2).

When comparing COVID-19 ARDS patients with HC, stimulation of PBMC by the overlapping S peptide pool led to a strong significant production of the Th1 or effector cytokines IFN-γ, TNF-α and IL-2 in COVID-19 ARDS patients. More characteristic Th2 cytokines (IL-5, IL-13, IL-9 and IL-10) were also consistently detected, albeit at low levels. IL-4 and IL-21 could not be detected at all. IL-6 levels were not different between COVID-19 patients and HC. However, these results were difficult to interpret because mock stimulation already resulted in high IL-6 expression. Antigen-specific production of cytokines related to a Th17 response was also consistently detected; PBMC from COVID-19 ARDS patients produced significantly more IL-17A, IL-17F and IL-22 than HC.

In general, stimulation of PBMC from COVID-19 ARDS patients with MP led to a dominant production Th1 or effector cytokines (IFN-γ, TNF-α, IL-2), but Th2 (IL-5, IL-13, IL-9, IL-10) and Th17 (IL-17A, IL-17F and IL-22) cytokines could also be detected. Although not enough COVID-19 ARDS patients were included in this study to correlate specific T cell responses to clinical outcome, we did observe differences in cytokine production profiles on a case-per-case basis (Fig. S2D). Plotting the respective cytokine quantities as a percentage of total cytokine production showed that either IL-6 (case 3, 5 and 9), TNF-α (case 1, 3 and 9), IL-2 (case 8) or IFN-γ (case 2, 4, 6, 7 and 10) dominated the response.

### Longitudinal detection of SARS-CoV-2-specific T-cell responses

Finally, we studied the kinetics of development of virus-specific humoral and cellular immune responses in COVID-19 ARDS patients included in this study. Real time RT-PCR detection of SARS-CoV-2 genomes in respiratory tract samples showed a decreasing trend over time (Fig. 6A, ANOVA repeated measures *p*<0.001), whereas virus-specific serum IgG antibody levels, measured by RBD ELISA, showed a significant increase (Fig. 6B, ANOVA repeated measures, *p*<0.001). Concomitantly, SARS-CoV-2-specific CD4^+^ and CD8^+^ T cells were detected in all patients at multiple time points. For CD4^+^ T cell responses, the frequencies of virus-specific responder cells increased significantly over time (Fig. 6C, ANOVA repeated measures, *p*<0.001), for CD8^+^ T cells this increase was not as apparent (Fig. 6D, ANOVA repeated measures, p=0.1001). We found evidence for a direct negative correlation between viral loads and IgG ELISA (*r*=0.6630, *p*<0.0001) and viral loads and CD4^+^ Tcells (*r*=0.5675, *p*=0.0007), and a positive correlation between the appearance of IgG antibodies and virus-specific T cells (*r*=0.6360, *p*=0.0002) (Fig. S3).

## DISCUSSION

Collectively, these data provide information on the phenotype, breadth and kinetics of virus-specific cellular immune responses in COVID-19 ARDS patients. We provide evidence that SARS-CoV-2-specific CD4^+^ and CD8^+^ T cells appear in blood of ARDS patients in the first two weeks post onset of symptoms. It is important to mention that this study focused on PBMC samples, but tissue-resident T cells undoubtedly play an important role in this early response. SARS-CoV-2-specific CD4^+^ T cells in blood typically had a central memory phenotype, whereas CD8^+^ T cells had a more effector phenotype. Peng *et al*. also identified HLA-B*40:01-restricted T cells with mainly a central and effector memory phenotype (*17*). Consistent production in response to viral antigen of IFN-γ, TNF-α, IL-2, IL-5, IL-13, IL-9, IL-10, IL-17A, IL-17F and IL-22 was observed, with a dominant production of the effector and Th1 cytokines. Due to limitations in the number of PBMC that could be obtained from severe COVID-19 ARDS patients in an ICU setting, we could not resolve which cells were responsible for production of which cytokine by intracellular cytokine staining.

Elevated levels of IL-6 in patient plasma have been correlated to respiratory failure in COVID-19 patients (*18*). Although we could not detect increased specific production of IL-6 in PBMC stimulated with peptide pools due to high background production in controls, we detected a dominant IL-6 and TNF-α response in cell culture supernatants from the patient deceased due to respiratory failure (case 3, Fig. S2D). To determine the role of T cells in COVID-19, it is crucial that the cell types responsible for the production of IL-6 and the concomitant ‘cytokine storm’ are identified in large comparative cohort studies.

We included PBMC obtained from ten buffy coats obtained before the SARS-CoV-2 pandemic as negative HC. These HC were similar to the studied COVID-19 ARDS patients regarding age and gender. In some instances, reactive T cells were detected in HC after MP stimulation, both on basis of T-cell activation and cytokine production (Fig. 4, 6 and 7). Since PBMC from these HC could not contain SARS-CoV-2-specific T cells, we hypothesize that these responses were cross-reactive and had been induced by circulating seasonal ‘common cold’ coronaviruses. If we consider samples with a SI > 3 as responders, we identified 2 out of 10 HC (20%) to have these cross-reactive T cells. Our study reports responses in unexposed individuals in the Netherlands. This fits well with the report of Grifoni *et al*. from the USA (*8*) and from Braun *et al*. from Germany (*19*), Le Bert *et al*. from Singapore (*20*), and Meckiff *et al*. from the UK (*21*), who all report significant rates of reactivity from unexposed subjects. Interestingly, Peng *et al*. did not see significant responses potentially reflecting geographical and temporal variations, or the importance of experimental conditions (*17*). It is possible that HLA genotypes influence these responses, as well as the SARS-CoV-2-responses that were detected in ARDS patients. This is a topic that merits further investigation. The role of preexisting SARS-CoV-2-reactive T cells as a correlate of protection or pathology is unclear, and needs to be addressed in prospective studies.

Novel SARS-CoV-2 vaccines are currently in development and mainly focus on the surface glycoprotein S as an antigen for efficient induction of virus-specific neutralizing antibodies. We now show that S can also be a potent immunogen for inducing virus-specific CD4^+^ and CD8^+^ T cells. This is in good concordance with publications on related coronaviruses SARS-CoV and MERS-CoV (*9*, *10*), and also with recent reports detecting SARS-CoV-2-specific T cell responses (*7*, *8*, *17*, *19*, *22*, *23*). Our study adds to that body of literature, as we specifically studied a well-defined ARDS patient cohort and studied samples longitudinally, while correlating these to viral loads, humoral responses, memory phenotypes and cytokine response profiles.

Here, we specifically studied T-cell responses in ARDS patients admitted to the ICU. By definition these are all severe COVID-19 patients, therefore we cannot draw any conclusions on how the T-cell responses relate to disease severity. Whether presence and certain phenotypes of T cells are correlated to a ‘good’ or ‘bad’ prognosis remains to be determined. Collectively, these data shed light on the potential variations in T-cell responses as a function of disease severity, an issue that is key to understanding the potential role of immunopathology in the disease, as well as to inform vaccine design and evaluation.

## MATERIALS AND METHODS

### Study design

Here, we set out to detect and characterize SARS-CoV-2-specific CD4^+^ and CD8^+^ T-cell responses in longitudinal PBMC samples obtained from COVID-19 ARDS patients. The patient cohort was well-characterized, including ten patients, and defined by a positive RT-PCR on a sample from the respiratory tract. From each patient, samples at multiple time points (day 0, 7, 14 and later if available) were tested. These patients were directly compared with ten HC.

This study relied on the use of pre-designed peptide MP containing overlapping peptides or predicted epitopes for stimulation of PBMC. T-cell activation and phenotype were determined by flow cytometry, whereas cytokine production was determined by a beads-based multiplex assay. Each stimulation assay consisted of 8 conditions: stimulation with 4 different MP, a negative DMSO control, a negative medium control, a positive PHA control and a CMV control. A sample nonresponsive to PHA stimulation would have been excluded from analysis (0 occurrences); all other data was included. Due to the limited nature of the material (PBMC from ARDS patients), activation after stimulation was measured in single determinations. All raw data obtained is provided in tabular format in Table S1.

### Ethical statement

Patients admitted into the intensive care unit (ICU) with Acute Respiratory Distress Syndrome (ARDS) resulting from SARS-CoV-2 infection at Erasmus MC, Rotterdam, the Netherlands were included in a biorepository study aimed at ARDS and sepsis in the ICU. The first EDTA blood samples for PBMC isolation were obtained no more than 2 days after admission into the Erasmus MC ICU. Samples were collected weekly until a final sample at 28 days post study inclusion or for as long as the patient was in the ICU. Patient care and research were conducted in compliance within guidelines of the Erasmus MC and the Declaration of Helsinki. Due to the clinical state of most ARDS patients (*i.e.* intubated, comatose), deferred proxy consent was obtained instead of direct written informed consent from the patients themselves. Retrospective written informed consent was obtained from patients after recovery. The study protocol was approved by the medical ethical committee of Erasmus MC, Rotterdam, the Netherlands (MEC-2017-417 and MEC-2020-0222). Healthy control (HC) human buffy coats were requested as a comparator group at the Sanquin Blood Bank (Rotterdam, the Netherlands); written informed consent for research use was obtained. HCs were slightly younger than the included COVID-19 patients, however this was a non-significant difference and we therefore consider the HC and COVID-19 patients age-matched.

### Diagnosis

Real-time RT-PCR on the E-gene was performed as described previously (*24*) on RNA isolated from sputa, nasopharyngeal or oropharyngeal swabs by MagnaPure (Roche Diagnostics, The Netherlands) using the total nucleic acid (TNA) isolation kit.

### PBMC isolation

PBMC were isolated from EDTA blood samples. Tubes were centrifuged at 200*g* for 15 min to separate cellular parts. The plasma-containing fraction was collected, centrifuged at 1200*g* for 15 min, and the plasma was aliquoted and stored at -20°C. The cellular fraction was reconstituted with phosphate-buffered saline (PBS) and subjected to Ficoll density gradient centrifugation (500*g*, 30min). PBMC were washed and frozen in 90% fetal bovine serum (FBS) and 10% dimethyl sulfoxide (DMSO, Sigma Life Science) at -135°C. Upon use, PBMC were thawed in IMDM (Lonza, Belgium) supplemented with 10% FBS, 100 IU of penicillin/ml, 100 μg of streptomycin/ml (Lonza, Belgium) and 2 mM L-glutamine (Lonza, Belgium) (I10F medium). PBMC were treated with 50 U/ml Benzonase (Merck) for 30 min at 37°C prior to use in stimulation assays.

### Epitope MegaPool (MP) design and preparation

SARS-CoV-2 virus-specific CD4 and CD8 peptides were synthesized as crude material (A&A, San Diego, CA), resuspended in DMSO, pooled and sequentially lyophilized as previously reported (*25*). SARS-CoV-2 epitopes were predicted using the protein sequences derived from the SARS-CoV-2 reference sequence (GenBank: MN908947) and IEDB analysis-resource as previously described (*11*, *26*). Specifically, CD4 SARS-CoV-2 epitope prediction was carried out using a previously described approach in Tepitool resource in IEDB (*27*, *28*) similarly to what was previously described (*11*), but removing the resulting Spike glycoprotein epitopes from this prediction (CD4-R(remainder) MP, n=246). To investigate in depth Spike-specific CD4^+^ T cells, overlapping 15-mer by 10 amino acids have been synthesized and pooled separately (CD-4 S(spike) MP, n=221). CD8 SARS-CoV-2 epitope prediction was performed as previously reported, using the NetMHCpan4.0 algorithm for the top 12 more frequent HLA alleles in the population (HLA-A*01:01, HLA-A*02:01, HLA-A*03:01, HLA-A*11:01, HLA-A*23:01, HLA-A*24:02, HLA-B*07:02, HLA-B*08:01, HLA-B*35:01, HLA-B*40:01, HLA-B*44:02, HLA-B*44:03) and selecting the top 1 percentile predicted epitopes per HLA allele (*11*). The 628 predicted CD8 epitopes were split in two CD8 MPs containing 314 peptides each.

### SARS-CoV-2 RBD ELISA

Serum or plasma samples were analyzed for the presence of SARS-CoV-2 specific antibody responses using a validated in-house SARS-CoV-2 receptor binding domain (RBD) IgG ELISA as previously described (*29*). Briefly, ELISA plates were coated with recombinant SARS-CoV-2 RBD protein. Following blocking, samples were added and incubated for 1 hour, after which the plates were washed and a secondary HRP-labeled rabbit anti-human IgG (DAKO) was added. Following a one hour incubation, the plates were washed, the signal was developed using TMB, and the OD_450_ was measured for each well. All samples reported here were interrogated for the presence of antibodies on the same plate.

### Ex vivo stimulations

PBMC were plated in 96-wells U bottom plates at 1 × 10^6^ PBMC per well in RPMI1640 (Lonza, Belgium) supplemented with 10% human serum, 100 IU of penicillin/ml, 100 μg of streptomycin/ml (Lonza, Belgium) and 2 mM L-glutamine (Lonza, Belgium) (R10H medium) and subsequently stimulated with the described CD4 and CD8 SARS-CoV-2 MPs at 1μg/ml. A stimulation with an equimolar amount of DMSO was performed as negative control, phytohemagglutinin (PHA, Roche, 1μg/ml) and stimulation with a combined CD4 and CD8 cytomegalovirus MP (CMV, 1μg/ml) were included as positive controls. Twenty hours after stimulation cells were stained for detection of activation induced markers and subjected to flow cytometry. Supernatants were harvested for multiplex detection of cytokines.

### Flow cytometry

Activation-induced markers were quantified via flow cytometry (FACSLyric, BD Biosciences). A surface staining on PBMC was performed with anti-CD3^PerCP^ (BD, clone SK7), anti-CD4^V450^ (BD, clone L200), anti-CD8^FITC^ (DAKO, clone DK25), anti-CD45RA^PE-Cy7^ (BD, clone L48), anti-CCR7^APC^ (R&D Systems, clone 150503), anti-CD69^APC-H7^ (BD, clone FN50) and anti-CD137^PE^ (Miltenyi, clone 4B4-1). T-cell subsets were identified via the following gating strategy: LIVE CD3^+^ were selected and divided in CD3^+^CD4^+^ and CD3^+^CD8^+^. Within the CD4 and CD8 subsets, memory subsets were gated as CD45RA^+^CCR7^+^ (naive, T_N_), CD45RA^-^, CCR7^+^ (central memory, T_CM_), CD45RA^-^CCR7^-^ (effector memory, T_EM_) or CD45RA^+^CCR7^-^ (terminally differentiated effectors, T_EMRA_). T cells specifically activated by SARS-CoV-2 were identified by up-regulation of CD69 and CD137. An average of 500,000 cells was always acquired, the gating strategy is schematically represented in (Fig. S1A-J). In analysis, PBMC stimulated with MP_CD8_A and MP_CD8_B were concatenated and analyzed as a single file for SARS-CoV-2-specific responses to MP_CD8.

### Multiplex detection of cytokines

Cytokines in cell culture supernatants from ex vivo stimulations were quantified using a human Th cytokine panel (13-plex) kit (LEGENDplex, Biolegend). Briefly, cell culture supernatants were mixed with beads coated with capture antibodies specific for IL-5, IL-13, IL-2, IL-6, IL-9, IL-10, IFN-γ, TNF-α, IL-17A, IL-17F, IL-4, IL-21 and IL-22 and incubated for 2 hours. Beads were washed and incubated with biotin-labeled detection antibodies for 1 hour, followed by a final incubation with streptavidin^PE^. Beads were analyzed by flow cytometry. Analysis was performed using the LEGENDplex analysis software v8.0, which distinguishes between the 13 different analytes on basis of bead size and internal dye. Quantity of each respective cytokine is calculated on basis of intensity of the streptavidin^PE^ signal and a freshly prepared standard curve.

### Statistical analysis

For comparison of CD3^+^ T cell percentages, CD4:CD8 ratios, CD69^+^CD137^+^ stimulated T cells and cytokine levels between HC and COVID-19 patients, all log transformed data was tested for normal distribution. If distributed normally, groups were compared via an unpaired *t* test. If not distributed normally, groups were compared via a Mann-Whitney test. Comparisons between different stimulations (DMSO versus MP) were performed by paired *t* test (normal distribution) or Wilcoxon rank test (no normal distribution). Two-tailed *p* values are reported throughout the manuscript. One-way ANOVA repeated measures was used to test for increasing or decreasing trends over sequential time points (0, 7 and 14 days post inclusion).

## Supplementary Materials

immunology.sciencemag.org/cgi/content/full/5/48/eabd2071/DC1 Figure S1. Flow cytometry gating strategy. Figure S2. SARS-CoV-2-specific cytokine production in COVID-19 ARDS patients. Figure S3. Correlations between kinetics of viral loads, virus-specific antibodies and virus-specific T cell responses. Table S1. Raw data (in Excel spreadsheet).
